# RNA as a genome architect: G-loops in G-quadruplex regulation

**DOI:** 10.1186/s40779-025-00683-3

**Published:** 2026-01-14

**Authors:** Jie Wang, Zhao-Jie Lyu, Qi Zhang, William C. Cho, De-Chao Feng

**Affiliations:** 1 Urology & Nephrology Center, Department of Urology, Zhejiang Provincial People’s Hospital (Affiliated People’s Hospital), Hangzhou Medical College, Hangzhou, 310014 China; 2 Department of Urology, Institute of Urology, West China Hospital, Sichuan University, Chengdu, 610041 China; 3 Department of Urology, Institute of Precision Medicine, Peking University Shenzhen Hospital, Shenzhen, Guangzhou, 518036 China; 4 Department of Clinical Oncology, Queen Elizabeth Hospital, Kowloon, Hong Kong SAR China; 5 Division of Surgery & Interventional Science, University College London, London, W1W 7TS UK

**Keywords:** G-quadruplex, RNA-DNA hybrids, Genome instability, DNA repair, Chromatin architecture

For decades, DNA G-quadruplexes (G4s) have been recognized as non-canonical secondary structures enriched in regulatory genomic regions, influencing transcription, replication, and genome stability [1, 2]. While their formation is predictable, the mechanisms governing their dynamic resolution in vivo have remained elusive. The recent work by Sato et al. [3] provides a transformative answer, elucidating a complete RNA-driven regulatory cycle termed the “G-loop” pathway. The authors delineate a sophisticated process where a nascent RNA transcript invades the DNA duplex opposite a folded G4, forming a stable RNA–DNA hybrid structure (the G-loop), a process facilitated by heterogeneous nuclear ribonucleoprotein A1 (hnRNPA1) and mediated by BReast-CAncer susceptibility gene 2 (BRCA2) and RAD51. Crucially, they uncover the disassembly mechanism: the dedicated helicases the DEAH-box helicase 36 (DHX36) and the Fanconi Anemia Group J (FANCJ), first unwind the G4, followed by cleavage of the RNA strand by the XPF-ERCC1 endonuclease, allowing DNA synthesis to restore the duplex. This entire cycle is orchestrated in a damage-independent manner, co-opting the ataxia-telangiectasia and RAD3-related kinase/ataxia telangiectasia mutated (ATR/ATM) checkpoint machinery. A pivotal finding is the role of RNA concentration as a molecular switch; stoichiometric RNA promotes resolution, while excess RNA stabilizes the G-loop, effectively locking the G4. This creates a direct feedback loop where transcriptional output directly dictates DNA conformation, with ablation of *Dhx36* or *Fancj* leading to G4/R-loop accumulation, transcriptional dysregulation, and genome instability. Notably, this understanding is further enhanced by the two-tier G4 suppression mechanism, which ensures genome stability. The first tier involves RNA-driven G-loop assembly, where RNA invasion promotes the unwinding of G4 structures, preventing the accumulation of harmful G4s. If this mechanism fails, G4s may persist into the S-phase, causing replication fork stalling and DNA double-strand breaks, leading to genomic instability. The second tier occurs during DNA replication, where helicases DHX36 and FANCJ resolve G4s encountered by replication forks, preventing replication stress and maintaining genome integrity. Figure 1 elucidates the G-loop concept.

**Fig. 1:**
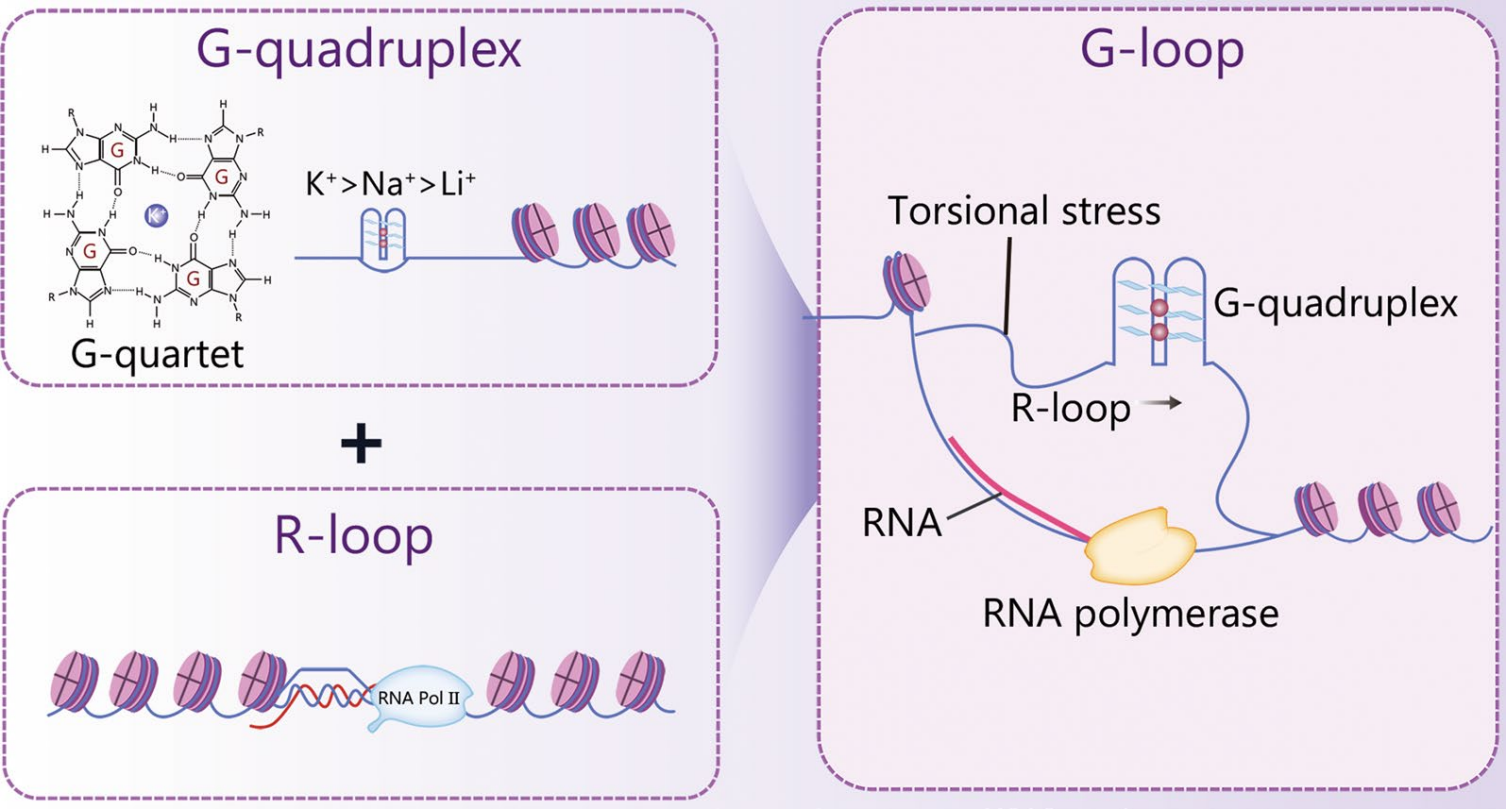
The concept of G-loop. G-quadruplex structures arise from guanine-rich DNA capable of folding into stacked G-quartets in the presence of monovalent cations (K⁺ > Na⁺ > Li⁺). R-loops form when nascent RNA invades the DNA duplex, creating an RNA-DNA hybrid and a displaced ssDNA strand. A G-loop is generated when an RNA-DNA hybrid forms opposite a G-quadruplex. ssDNA single-stranded DNA

The implications of this G-loop extend far beyond the mechanistic elegance of G4 resolution. It provides a mechanistic missing link between RNA biology and the genomic instability hallmark of cancer and neurodegenerative diseases. Proteins like BRCA2, RAD51, FANCJ, and XPF-ERCC1, with well-established roles in canonical DNA repair [4–6], are now implicated in managing endogenous structural threats posed by G4s. This suggests that defects in this pathway may be a hidden etiological factor in cancer predisposition syndromes associated with these genes. The G-loop model forces a re-evaluation of what constitutes “genome integrity”, expanding it to include the active maintenance of DNA secondary structure homeostasis.

Looking forward, the G-loop paradigm opens several frontier research avenues. First, it places RNA at the heart of chromosomal spatial organization. Could G-loops, by stabilizing specific G4s in promoter or enhancer regions, act as nucleation points for the formation of higher-order chromatin architectures and transcriptional hubs? Investigating the interplay between G-loops, chromatin modifiers and topologically associating domains could reveal principles of 3D genome folding driven by local DNA structure and RNA. Second, the discovery of RNA as a molecular switch presents a novel layer of metabolic regulation. Cellular nucleotide pools and transcription rates, which influence RNA abundance, could directly feed back onto G4 stability via the G-loop pathway. This connects cellular metabolic state to genome architecture, suggesting that metabolic dysregulation in diseases like cancer could exacerbate genomic instability through this mechanism. Furthermore, the potential for trans-acting RNAs to regulate G4s in cis or at distal genomic locations invites exploration of a vast, unexplored RNA-based regulatory network controlling DNA structure. Third, from a therapeutic perspective, the G-loop pathway is a treasure trove of potential targets. Small molecules that stabilize G4s (G4-ligands) have already been in clinical exploration [7]. The G-loop model suggests novel strategies: one could aim to disrupt the stabilizing factors of G-loops to resolve pathogenic G4 persistence, or conversely, to promote G-loop formation in oncogenic promoters to suppress gene expression. The specific involvement of DHX36, FANCJ and XPF-ERCC1 offers opportunities for synthetic lethal interactions in cancers with underlying G4 susceptibility or defects in complementary repair pathways. Finally, the role of G-loops in neural cells, which exhibit high transcriptional activity and are susceptible to G4-associated diseases like amyotrophic lateral sclerosis and frontotemporal dementia, demands urgent investigation [8]. The accumulation of G4s and R-loops in neurons could be directly linked through this pathway. Understanding whether G-loop resolution is compromised in neurodegeneration could uncover new pathomechanisms and therapeutic entry points.

In conclusion, Sato et al. [3] have delivered a milestone discovery that redefines RNA as an active guardian and architect of the genome. Their work unveils a fundamental principle: the genome is constantly patrolled for structural aberrations intrinsic to its own sequence and regulatory complexity. By delineating this RNA-driven surveillance cycle, they have laid the groundwork for a new era of exploration into RNA-chromatin interactions, with far-reaching implications for biology and medicine.

## Data Availability

Not applicable.

## Abbreviations

BRCA2: BReast-CAncer susceptibility gene 2
G4s: G-quadruplexes
DHX36: The DEAH box polypeptide 36
FANCJ: The Fanconi Anemia Group J
hnRNPA1: Heterogeneous nuclear ribonucleoprotein A1
